# Skin and Soft Tissue Infections in Patients with Solid Tumours

**DOI:** 10.1100/2012/804518

**Published:** 2012-02-01

**Authors:** Diamantis P. Kofteridis, Antonios Valachis, Eirini Koutsounaki, Sofia Maraki, Eleni Mavrogeni, Foteini N. Economidou, Dimitra Dimopoulou, Kostas Kalbakis, Vassilis Georgoulias, George Samonis

**Affiliations:** ^1^Infectious Disease Unit, Department of Internal Medicine, University Hospital of Heraklion, 71 110 Heraklion, Crete, Greece; ^2^Department of Clinical Microbiology, University Hospital of Heraklion, 71 110 Heraklion, Crete, Greece; ^3^Department of Medical Oncology, University Hospital of Heraklion, 71 110 Heraklion, Crete, Greece

## Abstract

*Background*. Skin and soft tissue infections (SSTIs) in cancer patients represent a diagnostic challenge, as etiologic diagnosis is often missing, and clinical assessment of severity is difficult. Few studies have described (SSTIs) in patients with solid tumours (STs). *Patients and Methods*. Records of patients with ST and SSTI, cared for at the University Hospital of Heraklion, from 2002 to 2006 were retrospectively studied. *Results*. A total of 81 episodes of SSTIs, occurring in 71 patients with ST, have been evaluated. Their median age was 65 years (34–82). The most common underlying malignancy was breast cancer in 17 patients (24%). Most episodes (89%) occurred in nonneutropenics. Cellulitis/erysipelas was the most common clinical presentation (56; 69%). Bacterial cultures were possible in 29 (36%) patients. All patients received antimicrobial therapy, while in 17 episodes (21%) an incision and drainage was required. Treatment failure occurred in 20 episodes (25%). Five patients (7%) died due to sepsis. None was neutropenic. Severe sepsis on admission (*P* = 0.002) and prior blood transfusion (*P* = 0.043) were independent predictors of treatment failure. *Conclusion*. SSTIs can be life threatening among patients with ST. Early diagnosis and appropriate treatment are of the utmost importance, since sepsis was proven a significant factor of unfavourable outcome.

## 1. Introduction

Despite the advances in prevention and management of infectious complications in cancer patients, they still remain an important cause of morbidity and mortality [1–4]. Additionally, infections, especially in neutropenic patients, may lead to delay in delivering planned chemotherapy, and this event may have an impact upon patients' survival [4–7].

Cancer patients are prone and highly susceptible to infections through obstructive processes or immune system disorders due to the disease per se or cytotoxic therapy and/or disruption of the integrity of anatomic barriers due to chemotherapy, radiotherapy, or intravascular devices [4, 8, 9].

Additionally, cancer patients are at risk of acquiring nosocomial infections, since they often undergo invasive diagnostic and therapeutic procedures, intravenous line placement, and hospitalization leading to the alteration of their skin and gut microbial flora [4, 10]. 

SSTIs in cancer patients represent a diagnostic challenge, as etiologic diagnosis is often missing, and clinical assessment of severity is difficult. Even small and innocuous in appearance, lesions should be carefully evaluated, because due to different degree of immunosuppression, the inflammatory response may be attenuated, and an apparently mild infection can become rapidly life threatening. Finally, SSTIs may be the result of a systemic disease spreading to the skin [4, 5, 11]. 

Therefore, early recognition and prompt administration of appropriate empirical antimicrobial treatment are crucial, especially in the neutropenic host [4, 5].

Few studies, however, have described SSTIs in immunocompromised patients, including those with cancer and neutropenia [7–12]. 

The purpose of our study was to describe the clinical and microbiological characteristics of SSTIs in patients with STs and to determine factors leading to treatment failure.

## 2. Materials and Methods

### 2.1. Setting

Records of patients with STs and SSTIs, cared for at the University Hospital of Heraklion, from 2002 to 2006 were retrospectively studied.

### 2.2. Ethical Approval

The study has been approved by the relevant committee of the University Hospital of Heraklion.

### 2.3. Study Design and Definitions

A SSTI was defined as community acquired if the episode developed in an outpatient setting or within 48 h after hospital admission in patients who did not fit the criteria for a healthcare-associated SSTI. Healthcare-associated SSTI was defined as the episode developing at the time of hospital admission or within 48 h after admission if the patient had been hospitalized or had been cared for at the day clinic of a hospital during the preceding 30 days [13]. Hospital-acquired SSTI was defined as the episode developing after the first 48 hours of hospital stay [13].

 SSTIs were grouped as cellulitis or erysipelas, furuncles or carbuncles, cutaneous abscesses, central venous catheter exit-site infection, wound infections, pyomyositis, and herpes zoster. Diagnoses were defined using established criteria [5].

Sepsis, severe sepsis, and septic shock were also defined using established criteria [14]. In more details, sepsis was defined as the clinical syndrome that results from a dysregulated inflammatory response to an infection. It exists if two or more of the following abnormalities are present, along with either a culture-proven or visually identified infection: temperature >38.5°C or <35°C, heart rate >90 beats/min, respiratory rate >20 breaths/min or PaCO2 <32 mmHg, WBC >12,000 cells/mm^3^, <4000 cells/mm^3^, or >10 percent immature (band) forms. 

Severe sepsis was defined as sepsis plus at least one of the following signs of hypoperfusion or organ dysfunction: areas of mottled skin, capillary refilling requiring three seconds or longer, urine output <0.5 mL/kg for at least one hour, or renal replacement therapy, lactate >2 mmol/L, abrupt change in mental status, abnormal electroencephalographic (EEG) findings, platelet count <100,000 platelets/mL, disseminated intravascular coagulation, acute lung injury or acute respiratory distress syndrome (ARDS), cardiac dysfunction (i.e., left ventricular systolic dysfunction), as defined by echocardiography or direct measurement of the cardiac index [14].

Patient's tumour response status to antitumour treatment at the date of onset of the SSTI episode was classified as complete response (CR), partial response (PR), stable disease (SD), or progressive disease (PD).

The definition of neutropenia was in accordance with the NCI common toxicity criteria, version 2.0 [15]. 

Empirical antimicrobial treatment was considered appropriate if the organism isolated from the infected area was susceptible at least to one of the antimicrobials prescribed. In any other case, treatment was considered inappropriate, on the condition that the SSTI had been attributed to a known organism.

Treatment failure was defined by documented worsening of clinical signs and symptoms of the infection, such as recurrence or worsening of fever, increased purulence, erythema, induration, and/or tenderness at least 2 days after treatment initiation, and if one of the following had happened: performance of a second, not previously planned, incision and drainage procedure, and/or readmission to the hospital due to the same SSTI episode and/or death due to septic shock.

### 2.4. Data Review

Patient's age, sex, underlying ST, blood transfusions, antimicrobial treatment, medications, exposure to radiotherapy or chemotherapy (during the preceding 30 days), and status of neoplastic disease (CR, PR, SD, PD), performance status (PS) and any invasive procedure performed (during the preceding 10 days), date of onset of the episode, date of previous admission, results of complete blood count (CBC) (total white blood cell count and differential, hemoglobin level, and platelets), presence of fever, hypotension, tachycardia, tachypnea, shock, signs of localized infection as well as antimicrobial treatment administered for every episode were systematically recorded.

Microbiological information included the results of all relevant cultures, the source and type of isolated pathogen(s), and its (their) susceptibility pattern(s).

### 2.5. Statistical Analysis

Bivariate analyses were conducted by Pearson's chi-square test and Fisher's exact test, to compare categorical variables. For continuous variables, *t*-test and nonparametric Mann-Whitney test (for not normally distributed variables) were used. Variables with a *P*-value <0.5 were subsequently included in a stepwise logistic regression multivariate analysis, in order to determine independent risk factors for treatment failure. The SPSS for Windows software, version 16.0 (SPSS Inc., Chicago, IL, USA), was used for all statistical analyses.

## 3. Results

### 3.1. Patient Characteristics

Eighty-one episodes of SSTIs occurring in 71 patients were identified. The median age of patients was 65 (range 34–82) years. There were 38 males (53.5%). The most common underlying malignancy was breast cancer in 17 patients (24%), followed by colon in 14 (20%) lung in 13 (18%), genital in 10 (14%), head and neck in 5 (7%), urinary tract in 4 (6%), and sarcoma in 3 (4%) (Table 1).

At the onset of the SSTI 3 patients had a CR (5%), 2 had a PR (2.5%), 29 had SD (38%), and 47 had PD (58%). At the time of the episode, 24 patients (34%) had a PS: 0, 41 ( 58%) PS:1, 12 (17%) PS: 2, and 4 (5.5%) PS 3.

Fifty out of 81 SSTI episodes (62%) were healthcare-associated, 25 (35%) community, and 6 (7%) hospital acquired.

A central venous catheter was present in 15 episodes (18.5%), blood had been transfused during the preceding 10 days in 21 (26%), and invasive procedure was recorded in 30 (37%). Chemotherapy and radiotherapy during the preceding 30 days had been administered in 52 (64%) and 19 (23.5%) episodes, respectively, and antimicrobial treatment in 30 (37%). 

Neutropenia was present only in 9 episodes (11%), being grade IV in 4, with median duration of 4 days (3–21). Factors potentially associated with the infectious episodes are summarized in Table 2.

### 3.2. Clinical Presentation

Cellulites/erysipelas were the most common presentations (44 episodes; 54%), followed by abscesses (18; 22%) and exit-site infections (8; 10%). Regarding the site of infection, SSTIs were more frequently located on the trunk (28; 35%) followed by the genital and/or perirectal region (19; 23%). 

Considering signs and symptoms, erythema was present in 59 episodes (70%), swelling in 41 (51%) and localized pain in 34 (42%). At the time of admission, fever was present in 52 (64%) with median duration 3.5 days (1–14). 

All the surgical site infections were wound infections while in all catheter-related infections, catheters had been removed.

Sepsis was present on admission in 43 episodes (53%) and severe sepsis in 15 (18.5%), while among the latter episodes 5 (4%) were complicated by septic shock. Clinical characteristics of the episodes are summarized in Table 3.

### 3.3. Microbiology

Pathogens were isolated from skin and/or soft tissue lesions in 29 episodes (36%). One pathogen was isolated from each of the 23 episodes, while two from the remaining 6. Gram-negative bacteria were isolated in 13 (45%), with *Escherichia coli* (6 out of 13; 46%) and *Pseudomonas aeruginosa* (4; 31%) being the most frequent, followed by *Enterobacter cloacae* (2; 15%) and *Klebsiella pneumoniae* (1; 8%). Gram-positive organisms were isolated in 9 episodes (11%) with *Staphylococcus epidermidis* being the most common (4 out of 9; 45%) followed by *Enterococcus faecalis* (3; 33%) and *Staphylococcus aureus* (2; 22%). The isolated pathogens isolated are shown in Table 4. 

Blood cultures were performed in all patients upon admission. No pathogens were isolated from blood.

### 3.4. Therapy

All patients received antimicrobial therapy, while in 17 episodes (21%) an incision and drainage were required. 

Initial antimicrobial treatment was considered inappropriate in 3 out of 20 episodes (15%) treated successfully and in 7 out of 9 (78%) treated unsuccessfully (*P* = 0.001).

### 3.5. Outcome and Predictors of Failure

Treatment failure occurred in 20 episodes (25%). An additional drainage procedure was necessary in 8. Seven episodes recurred with subsequent hospital admission and improved after treatment with second-line antimicrobial regimens. Five patients had a fatal outcome due to septic shock. Four of them have been inappropriately treated, and none was neutropenic. The overall mortality reached 7%. Characteristics of the 5 patients who died are shown in Table 5. 

In univariate analysis, three factors were found to have statistically significant association with treatment failure: prior blood transfusion, presence of severe sepsis on admission, and hospital-acquired or health-care associated infection. 

Multivariate logistic regression analysis indicated that presence of severe sepsis on admission (OR 21.921, 95% CI: 2.970–161.815; *P* = 0.002) and prior blood transfusion (OR 4.460, 95% CI: 1.049–18.954; *P* = 0.043) were independent predictors of treatment failure. 

## 4. Discussion

The present study showed that SSTIs can be life threatening in patients with ST even in the absence of neutropenia. The majority of SSTIs were health-care associated, with gram-negative bacteria being most commonly isolated. 

Cancer patients are at increased risk of infections, including SSTIs, due to multiple factors such as immunosuppression resulting from chemotherapy or due to the malignancy itself, impairment of normal leukocyte function, disruption of anatomical barriers, or obstructive phenomena [2–5, 7, 16]. 

SSTIs in patients with STs may have different etiology than those occurring in the general population and variable clinical presentations due to different degrees of immunosuppression and type of neoplasia. Additionally, a broad range of differential diagnoses that may mimic skin infections should be taken in consideration such as drug and transfusion-associated rashes, hemorrhagic areas, and malignancies.

In patients with tumours, a normally mild SSTI can be rapidly transformed into a life-threatening disease or can be a manifestation of a serious systemic disease spreading to the skin [4, 5]. Furthermore, SSTIs, especially in neutropenic patients, may delay the delivery of planned chemotherapy with impact on the outcome of the underlying malignancy [6].

Although infectious complications in patients with STs have been well described, specific data on SSTIs are rare and inconclusive [4, 5, 7–12]. 

The present results indicate that SSTIs can be life-threatening among patients with STs, even in the absence of neutropenia. Neutropenia remains an important risk factor for infection in patients with cancer although in the present study only a limited number of patients were neutropenic [2, 16, 17]. 

Additionally cancer patients are at increased risk of nosocomial infections since they often undergo invasive procedures, intravenous line placement, and hospitalizations leading to the alteration of their microbial flora [17]. Indeed the present study revealed that 62% of the episodes were health care associated while an additional 7% was pure nosocomial. This is not surprising considering the fact that most cancer patients nowadays, including the present ones, receive routine care in day clinics as outpatients [16, 17]. 

In the present study, cellulitis and erysipelas were the most common clinical presentations. Microorganisms were isolated from lesions in 36% of the episodes with gram-negative pathogens being the most common bacteria isolated and this is in contrast with other studies in the general population in which gram-positive organisms, *Staphylococcus aureus* and *Streptococcus pyogenes, *are the predominant pathogens [18, 19]. There are, however, studies in accordance with our results indicating that gram-negative bacteria play an important role as causative agents of skin and soft-tissue infections in many areas of the world, especially in patients with liver cirrhosis, malignancy, and alcoholism [20, 21].

It is worth noting that none of the 71 patients had positive blood cultures. Two recent literature reviews concluded that blood cultures are not necessary in acute cellulitis in immunocompetent hosts [22, 23]. Although the present results are negative, the question whether blood cultures are helpful in immunocompromised patients with SSTIs has not yet been answered [5]. 

The role of antimicrobial treatment of SSTIs is also not fully clarified. There are reports describing cures of immunocompetent patients receiving inappropriate or no treatment at all [24]. Furthermore, literature does not elucidate if inappropriate antimicrobial treatment in cancer patients with SSTIs plays a crucially negative role, considering also the fact that may delay the antitumour therapy due to prolonged infection [6]. However, in the present study, inappropriate initial antibiotic therapy was significantly associated with treatment failure. Additionally, four out of five patients with fatal outcome had received inappropriate treatment. 

Initial empirical antibiotic selection should be based on severity stratification of SSTIs and clinical care guidelines [5]. In stable pts with skin/soft tissue infections empiric therapy is usually aimed against gram-positive pathogens. However, as health-care-associated infections occur often among patients with solid tumors, empirical treatment with antimicrobial agents covering gram-negative bacteria should also be considered. 

Factors such as recent chemotherapy, prior invasive procedures, previous antimicrobial treatment, and prior blood transfusion are commonly associated with SSTIs in patients with STs [4, 5].

Multivariate analysis revealed that only two clinical factors had a statistically significant association with treatment failure: the occurrence of severe sepsis and the prior blood transfusion. 

Severe sepsis is an already known important complication in cancer patients developing infections that carries a high mortality rate [25]. The rate of sepsis occurring in the present patients was high. No other source except the SSTIs could be implicated for the septic episodes. This finding indicates the high risk of this type of infections in the present patients' population. 

The clinical significance of immune suppression secondary to blood transfusion in cancer patients remains controversial. Adverse effects of blood transfusions have been documented in patients undergoing colorectal and gastrointestinal surgery as demonstrated by higher infection rates among them [26, 27]. On the contrary, a meta-analysis that examined perioperative allogenic blood transfusion in surgical oncology patients concluded that there was no evidence to support adverse infectious sequelae [28]. 

SSTIs are usually curable [5]. However, in the present study, mortality reached 7%. A great number of the present population presented with severe and life-threatening infections. This can be explained by the fact that many infectious episodes were healthcare associated or nosocomial. 

Although 5 patients died due to septic shock that was related to some degree to their infection, no organisms were isolated from blood. It is of note that the disease's status and bad performance status did not affect significantly outcome. This is probably due to the fact that a relatively small number of patients had a PS ≥2. 

In conclusion, the majority of SSTIs in cancer patients of the present study were healthcare associated, with gram-negative bacteria being most commonly isolated from the infected sites. Furthermore, the study showed that SSTIs can be life threatening in this patients' population, even in the absence of neutropenia. Early diagnosis is of utmost importance, since sepsis on admission has been proven a significant factor of unfavourable outcome. Although 7% of the patients died due to septic shock that was related to some degree to their infections, no organisms were isolated from blood.

## Figures and Tables

**Table 1:** Patients' characteristics.

Characteristics	No of patients (%)
Sex of patients	
Male	38 (53.5%)
Female	33 (46.5%)
Median age of patients [range]	65 [34–82]
Underlying malignancy	
Breast	17 (24%)
Colorectal	14 (20%)
Lung	13 (18%)
Genital	10 (14%)
Head & neck	5 (7%)
Urinary tract	4 (6%)
Sarcoma	3 (4%)
Hepatobiliary	2 (3%)
Pancreas	1 (1.3%)
Stomach	1 (1.3%)
Unknown primary	1 (1.3%)

**Table 2:** Risk factors of the SSTIs episodes.

Factor	Episodes (%)
Recent chemotherapy	52 (64)
Prior invasive procedure	30 (37)
Previous antimicrobial therapy	30 (37)
Prior blood transfusion	21 (26)
Recent radiotherapy	19 (23.5)
Central venous catheter	15 (18.5)
Neutropenia	9 (11)

**Table 3:** Clinical characteristics of SSTI episodes.

Characteristic	No of episodes (%)
Type of SSTI	
Cellulitis/erysipelas	44 (54)
Abscess	18 (22)
Exit-site infection	8 (10)
Wound infection	5 (6)
Herpes zoster	4 (5)
Furuncles/carbuncles	1 (1)
Pyomyositis	1 (1)
Site of infection	
Head and neck	10 (12)
Trunk	28 (35)
Upper extremities	11 (14)
Lower extremities	13 (16)
Genitoperineal/perirectal	19 (23)
Signs and symptoms	
Erythema	59 (70)
Fever	52 (64)
Swelling	41 (51)
Localized pain	34 (42)
Sepsis	43 (53)
Severe sepsis	15 (18.5)
Septic shock	3 (4)

**Table 4:** Microorganisms isolated from 29 SSTIs episodes.

Pathogens	Total no of isolates
Gram negative	
* Pseudomonas aeruginosa*	7
* Escherichia coli*	7
* Klebsiella pneumoniae*	2
* Enterobacter cloacae*	2
* Citrobacter freundii *	1
Gram positive	
* Enterococcus faecalis*	6
* Staphylococcus epidermidis*	5
* Staphylococcus aureus*	3
* Enterococcus faecium*	1
Anaerobes	
*Bacteroides fragilis *	1

Total	35

**Table 5:** Characteristics of 5 patients with fatal outcome.

Age	Sex	Underlying malignancy	Type of infection	Isolated organisms	Absolute neutrophils count	Initial inappropriate therapy
76	F	Colorectal	Cellulitis	*Escherichia coli*	15000	Yes
64	M	Lung	Abscess	polymicrobial	18100	Yes
82	F	Breast	Abscess	negative	4700	—
48	M	Lung	Cellulitis	*Escherichia coli*	10100	Yes
56	M	Colorectal	Abscess	*Escherichia coli*	29500	Yes
